# Serendipitous Enhancement of the Dimensionality in Diketopyrrolopyrroles through O-Substitution

**DOI:** 10.1021/acs.cgd.2c01420

**Published:** 2023-01-20

**Authors:** Monika Warzecha, Alan R. Kennedy, Callum J. McHugh, Jesus Calvo-Castro

**Affiliations:** † EPSRC CMAC Future Manufacturing Research Hub, c/o Strathclyde Institute of Pharmacy and Biomedical Sciences, Technology and Innovation Centre, University of Strathclyde, 99 George Street, Glasgow G1 1RD, United Kingdom; ‡ Department of Pure & Applied Chemistry, University of Strathclyde, Glasgow G1 1XL, United Kingdom; § School of Computing, Engineering and Physical Sciences, University of the West of Scotland, Paisley PA1 2BE, United Kingdom; ∥ School of Life and Medical Sciences, University of Hertfordshire, Hatfield AL10 9AB, United Kingdom

## Abstract

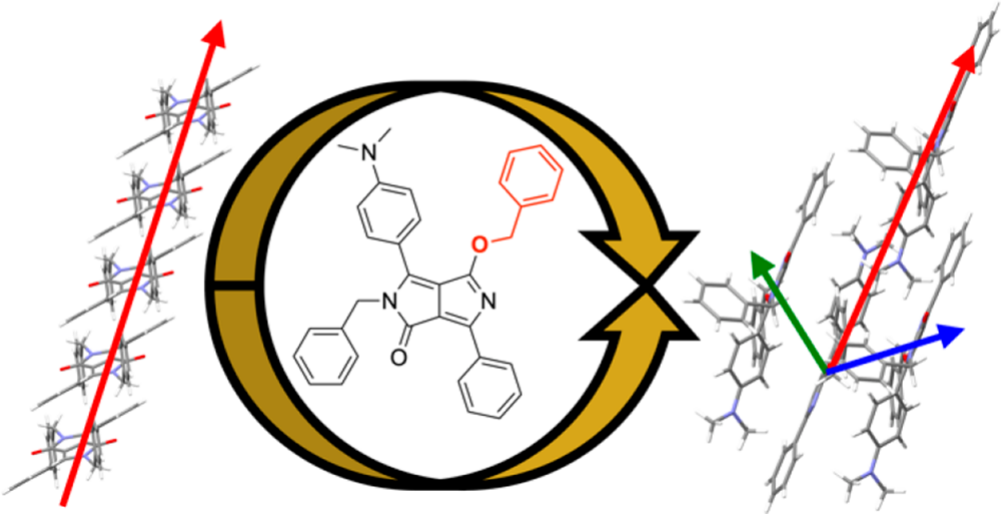

We report for the first time the crystal structure of an O-substituted diketopyrrolopyrrole and further evaluate computationally the ability of this higher-dimensionality system to act as a charge transfer mediator in optoelectronic devices.

## Abstract 2

We demonstrate that higher dimensionality can be accessed in diketopyrrolopyrroles through O-substitution when compared to their N,N′-disubstituted analogues.

The last two decades have seen increased interest toward the utilization of small organic conjugated materials as charge transport mediators, on account of their highly desirable properties and promising performance when compared to inorganic counterparts.^1^ More recently, emphasis has shifted toward design strategies that deliver consistent improvement and capitalize on theoretical material performance in organic field effect transistors (OFETs).^2,3^ Differences between theoretical (maximum) and experimental performance can be attributed to dynamic disorder, resulting in charges being localized and thus hampering charge transport phenomena. This is largely associated with the thermal integrity of supramolecular motifs and charge propagation channels as a result of unavoidable temperature-induced fluctuations in the equilibrium positions of atoms and molecules. As a result, there is a pressing need to develop novel materials employing judicious design strategies that diminish positional disorders ultimately leading to dynamic disorder.

In making novel organic architectures, two different approaches are often considered. The first is the exploitation of peripheral substitutions on core motifs for which successful performance has been observed. This has the added benefit of enhancing an understanding of structure–property relationships which are critical in facilitating the realization of superior structural analogues. The alternative to this is a more ambitious strategy to develop novel synthetic methodologies for the realization of previously unexplored core motifs, where motivations can be taken from existing systems.^4^ In addition, one could also consider a third approach whereby novel and/or existing chemistries are utilized toward implementing structural modifications on known core motifs and not on their periphery, essentially leading to architectures that may share optoelectronic properties resembling those of their parent motif. Amid the different chemistries that have been exploited toward the realization of small molecule organic charge transport mediators, diketopyrrolopyrroles (DPPs), which were widely used as high-performance pigments, are now ubiquitous in optoelectronic applications.^5^ This technological transition was facilitated through synthetic strategies whereby substitutions were performed on the lactam nitrogen atoms, hence disrupting strong intermolecular H-bonding interactions responsible for the insolubility of DPP pigments.^6^ From a synthetic perspective, low yields obtained from one-pot, base-catalyzed N,N-disubstitution have been associated with peripheral alterations unintentionally occurring at carbonyl oxygen and not the lactam nitrogen atoms.^7,8^ This competitive reactivity constitutes a serendipitous opportunity toward access of potentially superior DPP-based architectures. Accordingly, it has been demonstrated that O-alkylation denotes a successful strategy in the design of narrower bandgap systems while maintaining a lower-lying highest occupied molecular orbital (HOMO). This contrasts with most approaches where a reduction in the bandgap carries the undesired effect of raising the energy of the HOMO, which is further associated with decreasing both the short-circuit current (*J*_SC_) and open-circuit voltage (*V*_OC_) in photovoltaic systems.^9^

The overall performance of organic materials in optoelectronic applications requires both molecular as well as supramolecular engineering. To facilitate the translation of novel materials into commercial applications and define maximum experimental performance, it is common that the evaluation of their performance is carried out on organic single crystals. These denote an ideal platform in realizing effective performance in optoelectronic devices in light of their superior purity and longer-range order when compared against thin film counterparts.^4,5^ Unfortunately, despite significant efforts toward understanding the molecular behavior of O-substituted DPPs, the supramolecular self-assembly of such architectures remains unknown. Motivated by these observations and shortfalls, herein we report the first crystal structure of an N,O-disubstituted DPP architecture and evaluate its theoretical performance as an organic charge transfer mediator computationally. The new structure, **mDoBDPP**, was given a name in the form of **XYDPP**, in line with its topology (Figure 1A) where **m** and **o** indicate the asymmetric dimethylamino (**D**) and O-benzyl (**B**) substitution, respectively. It is of note that the latter induces significant structural changes when compared to “traditional” DPPs where both lactam nitrogen atoms are substituted (N,N′-disubstitution).^5^ The new crystal structure was serendipitously accessed from DMC/hexane (1:1) by slow evaporation of a cooled solution of its N,N′-disubstituted analogue (mDMADPP in the original publication),^10^ where we hypothesize it was present as an impurity, hence denoting further evidence of unintentional peripheral alternations in diketopyrrolopyrroles whereby the substitution occurs on the carbonyl oxygen(s) and not on the originally intended lactam nitrogen atom(s).

**Figure 1:**
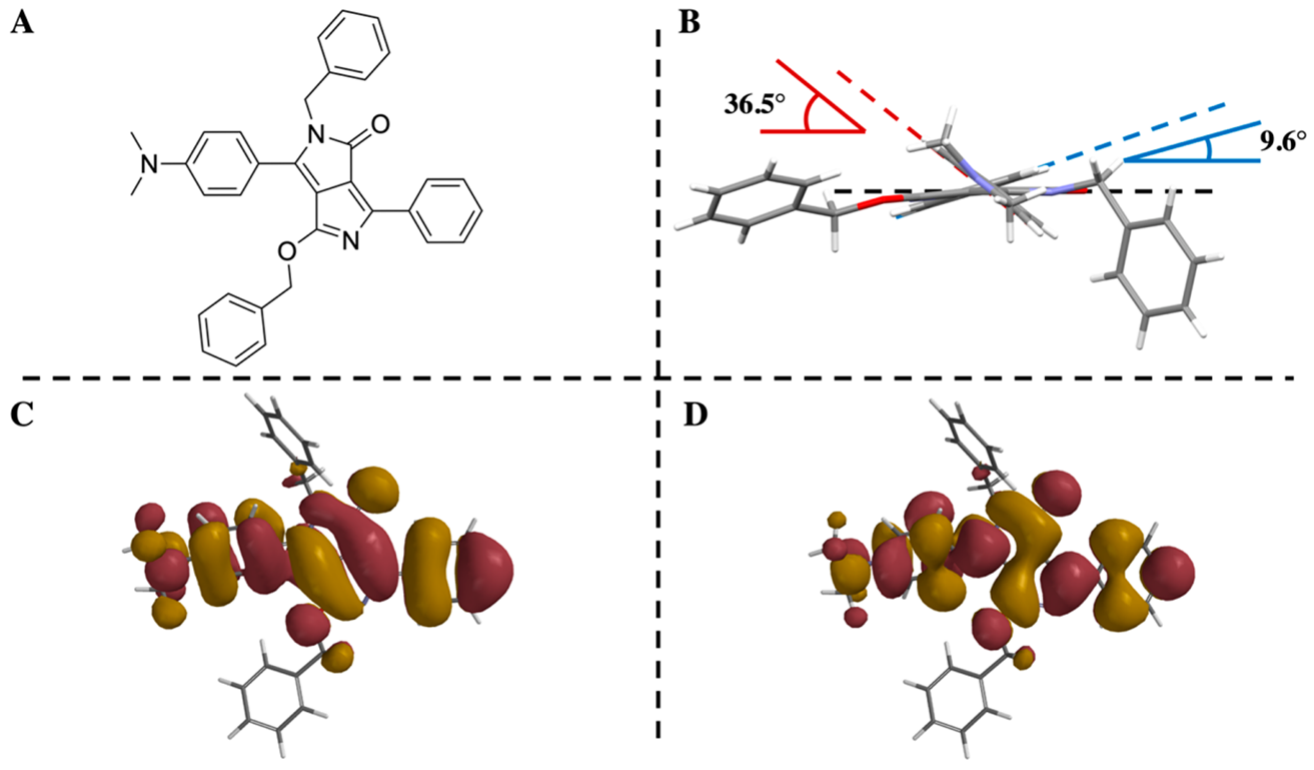
Chemical structure (A) and crystal structure viewed along the long molecular axis (substituted and nonsubstituted core rings dihedrals illustrated in red and blue, respectively) (B) and computed frontier molecular orbitals, HOMO (C) and LUMO (D), for **mDoBDPP**. For ease of interpretation, the orientation of **mDOBDPP** in panels C and D is that of the chemical structure in panel A.

The nonsubstituted core phenyl ring in **mDoBDPP** adopts an fairly coplanar (θ = −9.6(8)) arrangement with respect to the DPP core, comparable to the planarity observed in DPP pigments as well as those bearing mono N-substitution.^11−17^ In turn, the proximity of both O- and N-substitutions to the dimethylamino-bearing core phenyl ring induces a ca. −36.1(8)° out-of-plane rearrangement of this moiety (Figure 1B). This was observed to be greater than in the case of above-mentioned mono N-substituted DPP,^11−13^ which we attribute to the additional steric effect originating from the O-substitution in the structure reported herein. There is also a noteworthy structural difference in the relative orientation of the benzyl groups with respect to the core motif. While the phenyl group on the O-benzyl substituent adopts a quasi-planar relative orientation with respect to the DPP core, its N-benzyl counterpart exhibits a more “classical” arrangement where the phenyl group lies above/below the plane of the core motif (Figure 1B).^5,18,19^

When compared to “traditional” N,N′-disubstituted DPPs, **mDoBDPP** is characterized by a significantly different supramolecular motif. First, of note is the large number of nearest neighboring dimer pairs (13), which is greater than those observed in previously reported DPP-based supramolecular architectures.^18−20^ This is particularly relevant in light of the unavoidable thermal-induced fluctuations in organic semiconductors and the role of thermal integrity in preserving self-assembled supramolecular motifs. In addition, it is widely accepted that larger numbers of short contacts enhance the accessible charge transfer pathways in organic semiconductors.^2^ To further evaluate this characteristic in **mDoBDPP**, we computed the intermolecular interactions for all nonequivalent nearest neighboring dimer pairs as well as a series of systematically cropped versions (Table 1). Unlike other DPP-based architectures,^18−22^ we report large (>30 kJ mol^–1^) attractive energies for a number of the identified dimer pairs, namely, II, III, V, VIII (and Δ*E*_CP_ = −29.05 kJ mol^–1^ for dimer pair X). Dimer pairs II, VIII, and X exhibit comparable computed Δ*E*_CP_ values (Table 1) arising from supramolecular interactions previously observed in analogous DPP systems bearing benzyl groups on the lactam nitrogen atoms.^18−22^ While the asymmetry of the newly reported **mDoBDPP** precludes the self-assembly into “classical” slipped cofacial dimeric motifs, we observed that dimer pair II is characterized by a head-to-tail interaction whereby the top monomer is displaced with respect to the bottom one by 14.46 and 1.68 Å, along the long and short molecular axes, respectively (Figure 2). Thus, the Δ*E*_CP_ = −33.57 kJ mol^–1^ can be accounted for by means of the intermolecular interaction involving the *p*-dimethylamino substituted core rings. Notably, we observe a close interaction between neighboring amino methyl hydrogen and nitrogen atoms at a distance of 2.83 Å, and between the amino methyl groups with the O-benzyl aromatic ring (C···C distance 3.344(8) Å). Removal of these substituents from the monomers results in a complete cancellation of the computed Δ*E*_CP_ (Table 1). A comparable (Δ*E*_CP_ = −33.99 kJ mol^–1^) interaction energy was computed for dimer pair VIII. This can be attributed to a close T-shape interaction that engages the unsubstituted core phenyl rings and the aromatic substituents within the N-benzyl motifs (Figure 2). We observed that removal of the latter on the cropped dimer pairs results in an almost complete (95.5%) cancellation of the computed interaction. On the other hand, removal of the core phenyl rings was observed to lower that computed interaction by 53.2% instead. This can be accounted for on the basis of the additional attractive H-bonding interaction involving one of the *m*-phenylic hydrogen atoms within the N-benzyl group and the carbonyl oxygen atom at 2.62 Å. The computed interaction (Δ*E*_CP_ = −29.05 kJ mol^–1^) for dimer pair X is also attributed to a H-bonding interaction. In this case, it involves the electropositive N-benzyl methylene hydrogen atoms and the electropositive carbonyl oxygen atoms at 2.56 Å in this dimer pair. Through the analysis of the cropped analogous dimer pair, it was confirmed that removal of the N-benzyl substituent results in a 67.4% reduction in the overall computed energy. The remaining stability can be attributed to a weak interaction between the unsubstituted core rings and the O-benzylic aromatic ring.

**Table 1:** Computed Hole (*t*_h_) and Electron (*t*_e_) Transfer Integrals and Counterpoise-Corrected Intermolecular Interactions (Δ*E*_CP_) for All Nearest Neighboring Dimer Pairs of **mDoBDPP** as Well as Their Structurally Related Analogues^a^

			Δ*E*_CP_ (structurally modified dimer pairs)/kJ mol^–1^				
dimer pair	t_h/e_/meV	Δ*E*_CP_**/**kJ mol^–1^	A	B	C	D	core
I	<0.1/<0.1	–22.92	–1.93	–8.63	–20.04	–22.27	–0.07
II	8.1/1.7	–33.57	0.00	–33.57	–32.38	–24.78	0.31
III	10.4/13.9	–92.28	–40.16	–83.03	–85.65	–40.63	0.37
IV	4.1/2.8	0.25	0.31	0.63	0.20	0.27	0.20
V	7.0/0.2	–45.79	–29.48	–44.61	–43.71	–6.23	–0.59
VI	<0.1/< 0.1	–4.56	–6.1	–3.94	0.64	0.81	–0.47
VII	1.3/1.2	–13.31	–13.68	0.54	–13.39	0.17	0.08
VIII	1.2/1.7	–33.99	–31.78	–15.91	–1.53	–34.29	–1.24
IX	<0.1/<0.1	–14.74	–5.36	–4.64	–11.19	–14.42	–9.29
X	0.6/0.2	–29.05	–29.45	–23.53	–9.46	–29.07	–0.09
XI	<0.1/0.5	–14.87	–5.21	–14.77	–2.82	–14.96	–0.16

a Where letters A–D indicate the functional group cropped (i.e., substituted (A) and nonsubstituted (B) core phenyl ring, N-benzyl (C) and O-benzyl (D) groups) at M06-2X/6-311G(d).

**Figure 2:**
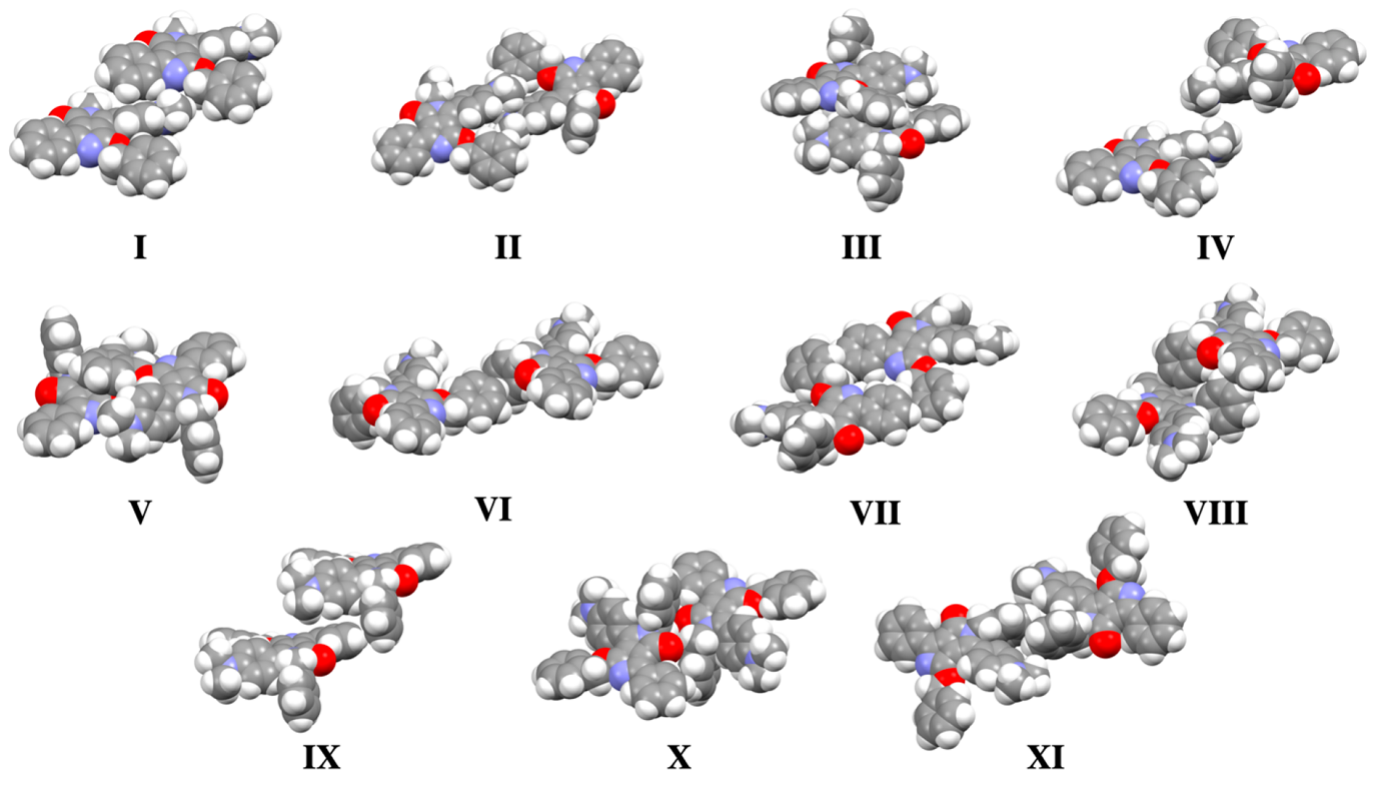
Space-filled illustration of all (I–XI) inequivalent nearest neighbor dimer pairs in **mDoBDPP**.

Dimer pairs III and V were in all cases observed to exhibit computed Δ*E*_CP_ dominated by intermolecular interactions involving the O-benzyl substituents (Table 1). In the case of dimer pair V, the interaction arises from a close π–π overlap of the O-benzyl ring with the DPP core, located ca. 3 Å apart. In fact, removal of the O-benzyl substituents on progression to the analogous structurally modified dimer pair leads to an 86.4% weaker dimeric interaction. Interestingly, we calculated the largest interaction energy (Δ*E*_CP_ = −92.28 kJ mol^–1^) for dimer pair III, despite the largest observed displacements (Δ_*x*/*y*_ = 7.63/4.86 Å). This highlights that that strong interactions are not solely restricted to DPP-based systems exhibiting close alignment along the short molecular axis.^5^ In this regard, the head-to-tail arrangement facilitates primarily the interaction of the O-benzyl substituents with the core ring as well as with the DPP core. Accordingly, removal of the dimethylamino substituted core ring results in a 56.5% decrease in the computed interaction energy, primarily associated with the disruption of a H-bonding interaction between the O-benzylic oxygen atoms and the *o*-phenylic hydrogens 2.85 Å apart. This is further supported by the analogous (56.0%) reduction in the computed interaction energy on removal of the O-benzyl moieties. The remainder of the overall computed interaction can be attributed to a pseudo-T-shape interaction engaging the aromatic core of the O-benzyl substituents and the core of the DPP as well as the interaction between the electropositive hydrogen atoms of the dimethylamino groups and the electronegative unsubstituted core rings. As a result, we anticipate an exceptional thermal integrity for **mDoBDPP**, for which we compute the largest intermolecular interaction of all DPP systems reported to date, exceeding that computed for our two-dimensional cruciform (Δ*E*_CP_ = −332.31 and −299.06 kJ mol^–1^ for **mDoBDPP** and the latter, respectively).

Next, in light of the role played by short contacts in facilitating charge transport, we evaluated the ability of these dimeric systems to act as charge transfer mediators.^2^ In most cases, the self-assembly of diketopyrrolopyrroles results in the generation of one-dimensional slipped cofacial stacking motifs, with transfer integrals approaching or even exceeding those computed for gold standard materials such as rubrene.^23^ We have previously demonstrated that double dimensionality can be accessed in DPPs via isosteric fluorine substitution.^21^ However, there is a requirement to develop design strategies that can access materials with even higher dimensionality. Table 1 summarizes the computed transfer integrals for hole (*t*_h_) and electron (*t*_e_) for all the inequivalent nearest neighbor dimer pairs of **mDoBDPP**. While we computed low (<2 meV) transfer integrals for dimer pairs VII, VIII, X, and XI, in line with their relative orientation, we note those computed for dimer pair IV. Despite the negligible structural overlap in this dimeric system (e.g., Δ*E*_CP_ = 0.25 kJ mol^–1^), we report *t*_h/e_ = 4.1/2.8 meV values which are comparable to those reported for one of the propagation channels in our previously reported fluorine-containing cruciform (*t*_h_/*t*_e_ = 2.65/7.90 meV).^21^ These can be attributed to weak bonding (HOMO – 1 and LUMO) and antibonding (HOMO and LUMO + 1) interactions between regions of the frontier molecular orbitals on the dimethylamino groups.

While large and comparable transfer integrals for both holes and electrons are often sought after for ambipolar behavior, superior hole transport behavior is desirable owing to inherent susceptibility of electrons to quenching.^24^ In this regard, progression from dimer pair IV to II results in a closer alignment of these terminal groups along both short (*y*) and *z* axes, leading to an increase/decrease of the hole/electron transfer integrals (*t*_h/e_ = 8.1/1.7 meV). The larger *t*_h_ can be ascribed to a moderate bonding interaction of the HOMO wave function, while that in the HOMO – 1 is antibonding in nature (Figure 3). A similar trend was observed for dimer pair V, also characterized by larger hole than electron transfer integrals (t_h/e_ = 7.0/0.2 meV). In this case, the computed *t*_h_ was solely accessed due to the O-benzyl substitution in **mDoBDPP**, which facilitates a significant HOMO density on to the benzyl group, unlike with N-substitution.^5^ In line with the computed intermolecular interaction for dimer pair III (Table 1), we also computed the largest transfer integrals for this architecture (*t*_h/e_ = 8.1/1.7 and 10.4/13.9 meV for dimer pairs II and III, respectively). Despite the significant decrease in long molecular axis slip on going from dimer pair II to III, we observe just a moderate increase in *t*_h_. This can be attributed to the large displacement along the short molecular axis in the latter, which results in only moderate bonding/antibonding HOMO/HOMO – 1 interactions. Thus, this contrasts with the strong bonding/antibonding nature of analogous interactions in dimer pair II, albeit only involving the terminal motifs which accounts for the computed values.

**Figure 3:**
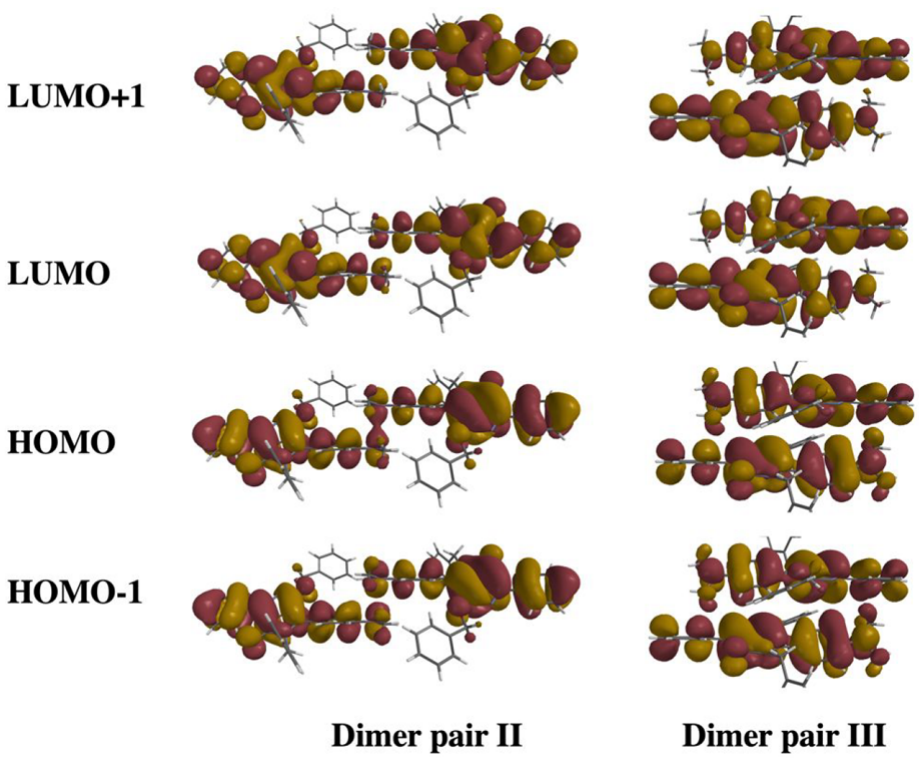
Illustration of the supramolecular orbitals for the dimer pairs II and III (N-benzyl groups cropped for clarity) of **mDoBDPP**. IsoVal = 0.01.

We devote the remainder of this communication to the evaluation of the computed inner-sphere reorganization energies in **mDoBDPP** for both hole (λ_h_) and electron (λ_e_) transfer processes, which further denotes an important parameter within the hopping regime formalism for charge transport in organic materials. We report total (i.e., sum of neutral and radical contributions) inner-sphere reorganization energies for **mDoBDPP** of 49.62 and 29.86 kJ mol^–1^ for hole and electron transfer, respectively, which are in line with those previously determined for N,N′-disubstituted DPPs.^25^ However, in-depth analysis of the optimized geometries for neutral and radical species revealed that the nature of such relative order in N,O-disubstituted DPP when compared to “traditional” N,N′-disubstituted analogues obeys a completely different structural rearrangement. In short, while λ_h/e_ can be primarily rationalized in the latter based on torsional as well as out-of-plane motions of both core rings with respect to the central motif, these rearrangements were no longer able to account for the computed values for **mDoBDPP**. We observed that progression from neutral to optimized radical cation geometry results in a structural rearrangement, absent in the radical anion counterpart whereby the O-benzylic phenyl ring adopts an almost perpendicular orientation with respect to the DPP core. As a result, we confirm that the different substitution in N,O-disubstituted DPPs does not have the associated detrimental impact of higher inner-sphere reorganization energies when compared to their N,N′-disubstituted counterparts.

In conclusion, progression from “traditional” N,N′-disubstituted diketopyrrolopyrroles to their O-substituted counterparts can serve as a design strategy to increase highly sought-after dimensionality in this class of materials. The reported structure self-assembles into a supramolecular motif giving rise to a number of efficient charge propagation channels while retaining comparable inner-sphere reorganization energies when compared to N,N′-disubstituted analogues. In addition, a large thermal integrity of these channels is anticipated in light of the computed intermolecular interactions, where the O-benzyl substituted species plays a significant role. As a result, we hope that this work will further stimulate research in this area, in the quest to realize superior organic charge transfer mediating materials exploiting diketopyrrolopyrrole chemistries.

## Supporting Information Available

Crystallographic information and computational details (PDF)
