# Supplementary material for: Serendipitous Enhancement of the Dimensionality in Diketopyrrolopyrroles through O-Substitution
Source: Cryst Growth Des. 2023 Jan 20;23(2):670–5. doi: 10.1021/acs.cgd.2c01420 (PMC9999410; doi:10.1021/acs.cgd.2c01420)
Supplement: Supplementary file 1 — cg2c01420_si_001.pdf [file cg2c01420_si_001.pdf]

Supplementary Information for:

Serendipitous Enhancement of the Dimensionality in Diketopyrrolopyrroles Through O-substitution

Monika Warzecha,[a] Alan R. Kennedy,[b] Callum J. McHugh[c] and Jesus Calvo-Castro*[d]*

[a] EPSRC CMAC Future Manufacturing Research Hub, c/o Strathclyde Institute of Pharmacy and Biomedical Sciences, Technology and Innovation Centre, 99 George Street, Glasgow, G1 1RD, UK.

[b] Department of Pure & Applied Chemistry, University of Strathclyde, Glasgow G1 1XL, UK.

[c] School of Computing, Engineering and Physical Sciences, University of the West of Scotland, Paisley, PA1 2BE, UK.

[d] School of Life and Medical Sciences, University of Hertfordshire, AL10 9AB, UK.

*Corresponding authors: j.calvo-castro@herts.ac.uk; callum.mchugh@uws.ac.uk.

		Page
**SI1**	**Crystallographic information**	**2-3**
**SI2**	**Computational details**	**4-13**
**SI3**	**References**	**14-15**

SI1 Crystallographic Information

Single Crystal Diffraction. Crystallographic data were measured with a with a Rigaku AFC12 instrument with monochromated Mo−Kα (λ 0.71073 Å) radiation by the UK National Crystallography Service.[1] The structure was refined with ShelXL-2018[2] to convergence against F^2. Final refinement was within WinGX.[3] All non-hydrogen atoms were refined using anisotropic thermal parameters and the H-atoms were modelled in riding modes. Selected crystallographic and refinement data is given in Table SI1.1.

Complete single crystal diffraction data are reported in the crystallographic information file (CIF) accompanying this document and deposited with the CCDC as 2223450.

Table SI1.1 Selected Crystallographic and Refinement Parameters.

**Compound**	**mDoBDPP**
**Formula**	$C_{34}H_{29}N_3O_2$
**Form. Wt.**	511.60
**Space Group**	P-1
**Crystal system**	triclinic
**Temp. (K)**	100(2)
**Wavelength (Å)**	0.71073
**a (Å)**	8.198(6)
**b (Å)**	11.541(9)
**c (Å)**	14.621(10)
**α (°)**	85.70(4)
**β (°)**	81.77(4)
**γ (°)**	71.81(2)

**Volume (Å^3)**	1300.0(17)
**Z**	2
**Measured Reflections**	13197
**Unique Reflections**	4082
**Obs. Reflections**	2079
**R_{int}**	0.1224
**2Θmax (°)**	48.0
**No. Parameters**	354
**S**	1.068
**R [on *F*, obs refs only]**	0.0924
**ωR [on F^2, all data]**	0.1870
**Largest diff. peak /hole (eÅ^{-3})**	0.285/-0.240

SI2 Computational Details

Intermolecular interactions, DE_{CP}. Intermolecular interactions were, in all cases determined employing the M06-2X[4] density functional at the 6-311G(d) level as implemented in Spartan '20 (v.1.1.1) software[5] and corrected for Basis Set Superposition Error (BSSE) by means of the counterpoise method of Boys and Bernardi.[6]

Charge transfer integrals, $t_{h/e}$. Transfer integrals for hole (t_h) and electron (t_e) were computed employing the M06-2X[4] density functional at the 6-311G(d) level as implemented in Spartan '20 (v.1.1.1) software.[5] Within the framework of the energy-splitting-in-dimer method, t_h and t_e can be equated to half the splitting between the HOMO/HOMO(-1) and LUMO/LUMO(+1) supramolecular orbitals, respectively.[7,8]

Inner-sphere reorganisation energies, $\lambda_{h/e}$. These were calculated within the framework of the four-point method, as the sum of the reorganisation energies on going from neutral to radical species and *vice versa*.[7–10] As such, $\lambda_{h/e}$ on progression from neutral to radical (λ_{NR}) was calculated by subtracting the energy of the neutral species at its equilibrium geometry to that of the neutral species at the equilibrium geometry of the radical. Similarly, $\lambda_{h/e}$ on going from radical to neutral (λ_{RN}) species was determined as the difference between the energy of the radical at its equilibrium geometry and the energy of the radical at the equilibrium geometry of the neutral. The geometries of neutral (restricted) and radical (unrestricted) species were optimised employing the M06-2X[4] density functional at the 6-311G(d) level as implemented in Spartan '20 (v.1.1.1) software.[5] In all cases, optimised geometries (Table SI2.1-3) were confirmed by IR analysis, characterised by the absence of imaginary modes, hence consistent with true equilibria minima. For all radical species, $S^2 < 0.77$, which indicates low spin contamination in all cases.[11,12]

Table SI2.1 Neutral geometry of **mDoBDPP**, optimised at M06-2X/6-311G(d) with absolute energy of -1628.126452 hartrees.

**Atom**	**x / Å**	**y / Å**	**z / Å**
**O**	-1.278729	-2.187579	-3.563312
**O**	0.469285	-1.323187	2.030279
**N**	-1.069268	-0.320484	-2.179802
**N**	0.392693	-3.215402	0.657431
**N**	-0.825111	5.008216	1.71246
**C**	-0.971811	-1.74149	-2.47267

**C**	-0.498995	-2.322027	-1.240709
**C**	-0.385455	-1.278958	-0.273805
**C**	-0.739971	-0.062593	-0.854966
**C**	0.185583	-1.927962	0.871289
**C**	-0.013836	-3.48771	-0.64563
**C**	-1.768498	0.547476	-3.11936
**H**	-2.384153	1.253409	-2.557998
**H**	-2.444761	-0.107352	-3.676931
**C**	-0.895641	1.292333	-4.117122
**C**	-1.387824	2.461968	-4.70491
**H**	-2.361643	2.843736	-4.407694
**C**	-0.646544	3.14509	-5.665477
**H**	-1.045974	4.050838	-6.11168
**C**	0.606143	2.669322	-6.04833
**H**	1.188523	3.200775	-6.794498
**C**	1.104713	1.506323	-5.466324
**H**	2.077398	1.125225	-5.760362
**C**	0.358123	0.820228	-4.509667
**H**	0.752353	-0.090075	-4.072555
**C**	-0.780111	1.252817	-0.23947
**C**	-0.481242	2.442492	-0.92919
**H**	-0.193711	2.408151	-1.97195
**C**	-0.484652	3.670747	-0.293907
**H**	-0.216802	4.545977	-0.870179

**C**	-0.810015	3.787438	1.078551
**C**	-1.115939	2.589958	1.770477
**H**	-1.375408	2.615754	2.820353
**C**	-1.091489	1.36705	1.128334
**H**	-1.327654	0.473033	1.691498
**C**	-0.368778	6.196619	1.009236
**H**	0.68196	6.122497	0.700619
**H**	-0.469284	7.060923	1.663258
**H**	-0.972331	6.387597	0.116954
**C**	-0.998797	5.07562	3.154953
**H**	-1.953941	4.640477	3.461892
**H**	-0.999441	6.118829	3.466378
**H**	-0.196556	4.558053	3.697304
**C**	0.110538	-4.833537	-1.185489
**C**	-0.319468	-5.136456	-2.491185
**H**	-0.752956	-4.357376	-3.108201
**C**	-0.190479	-6.427202	-2.98929
**H**	-0.52582	-6.647051	-3.997943
**C**	0.363403	-7.43684	-2.201698
**H**	0.461909	-8.443775	-2.595283
**C**	0.789347	-7.146976	-0.90495
**H**	1.220344	-7.929254	-0.287756
**C**	0.666309	-5.859307	-0.399698
**H**	0.994513	-5.621368	0.604436

**C**	1.066828	-2.151957	3.043851
**H**	1.975671	-2.605104	2.640195
**H**	0.378261	-2.971565	3.271206
**C**	1.3628	-1.331151	4.274172
**C**	0.801352	-0.072463	4.490738
**H**	0.168223	0.365517	3.730162
**C**	1.070495	0.62738	5.666198
**H**	0.628355	1.607151	5.820172
**C**	1.901314	0.079042	6.639076
**H**	2.108332	0.624774	7.553786
**C**	2.470505	-1.175791	6.426602
**H**	3.126422	-1.610465	7.174363
**C**	2.204694	-1.872418	5.251674
**H**	2.656698	-2.847958	5.092095

Table SI2.2 Radical anion geometry of **mDoBDPP**, optimised at M06-2X/6-311G(d) with absolute energy of -1628.180209 hartrees.

**Atom**	**x / Å**	**y / Å**	**z / Å**
**O**	-1.023976	-2.140168	-3.612322
**O**	0.374283	-1.351672	2.120332
**N**	-0.924732	-0.297966	-2.190511
**N**	0.387946	-3.231719	0.687647
**N**	-1.060543	5.080322	1.754307
**C**	-0.780496	-1.692729	-2.482396
**C**	-0.36884	-2.290715	-1.245216

**C**	-0.330295	-1.271252	-0.248796
**C**	-0.683843	-0.028608	-0.819499
**C**	0.162666	-1.94095	0.902288
**C**	0.078974	-3.487764	-0.636052
**C**	-1.71815	0.51292	-3.106437
**H**	-2.321023	1.21692	-2.529406
**H**	-2.401191	-0.177726	-3.611881
**C**	-0.93844	1.259004	-4.178144
**C**	-1.243433	2.588454	-4.483303
**H**	-2.013734	3.10173	-3.912899
**C**	-0.56801	3.267181	-5.497924
**H**	-0.81526	4.303043	-5.713614
**C**	0.42741	2.61836	-6.223485
**H**	0.961259	3.142895	-7.011316
**C**	0.734306	1.289305	-5.930731
**H**	1.504177	0.77353	-6.498124
**C**	0.057324	0.612271	-4.920008
**H**	0.268631	-0.430657	-4.71265
**C**	-0.757224	1.271601	-0.207113
**C**	-0.568527	2.490025	-0.897879
**H**	-0.301224	2.476286	-1.946968
**C**	-0.647394	3.722447	-0.262414
**H**	-0.468103	4.60868	-0.859253
**C**	-0.921562	3.829293	1.114703

**C**	-1.071932	2.615696	1.820548
**H**	-1.261388	2.618763	2.887375
**C**	-0.998164	1.389224	1.183754
**H**	-1.118963	0.488221	1.772006
**C**	-0.579949	6.251502	1.047244
**H**	0.502336	6.222541	0.832139
**H**	-0.785418	7.140491	1.647807
**H**	-1.105461	6.373548	0.096977
**C**	-0.880361	5.123177	3.194484
**H**	-1.620078	4.500565	3.700931
**H**	-1.03193	6.147309	3.541411
**H**	0.120218	4.791922	3.519046
**C**	0.231713	-4.818984	-1.188786
**C**	-0.105133	-5.107288	-2.530477
**H**	-0.489076	-4.310949	-3.160735
**C**	0.048857	-6.390359	-3.041588
**H**	-0.218389	-6.583134	-4.078113
**C**	0.538114	-7.428314	-2.244691
**H**	0.656168	-8.429639	-2.650493
**C**	0.87177	-7.156561	-0.915366
**H**	1.253389	-7.952352	-0.279138
**C**	0.722931	-5.878041	-0.394637
**H**	0.98082	-5.664851	0.636204
**C**	0.915003	-2.195644	3.124101

**H**	1.80997	-2.697013	2.736887
**H**	0.202987	-2.99267	3.370505
**C**	1.251569	-1.378165	4.348232
**C**	1.365921	0.013152	4.30075
**H**	1.167929	0.525269	3.367094
**C**	1.719677	0.732294	5.441947
**H**	1.802596	1.813686	5.386372
**C**	1.96822	0.074477	6.644594
**H**	2.243762	0.636861	7.532145
**C**	1.854533	-1.31397	6.700199
**H**	2.040435	-1.83998	7.632945
**C**	1.495401	-2.03047	5.561885
**H**	1.400954	-3.112649	5.615508

Table SI2.3 Radical cation geometry of **mDoBDPP**, optimised at M06-2X/6-311G(d) with absolute energy of -1627.906123 hartrees.

**Atom**	**x / Å**	**y / Å**	**z / Å**
**O**	-1.344872	-2.20065	-3.533406
**O**	0.441174	-1.403542	2.010972
**N**	-1.087237	-0.320405	-2.178181
**N**	0.331808	-3.298967	0.661868
**N**	-0.455922	4.936819	1.723555
**C**	-1.02015	-1.740002	-2.465729
**C**	-0.537868	-2.334661	-1.226769
**C**	-0.397897	-1.328162	-0.286693

**C**	-0.725876	-0.055392	-0.877791
**C**	0.161519	-2.002798	0.879494
**C**	-0.078388	-3.557801	-0.620964
**C**	-1.733495	0.556587	-3.159057
**H**	-2.377393	1.258273	-2.624657
**H**	-2.385445	-0.098844	-3.74155
**C**	-0.800925	1.293193	-4.10662
**C**	-1.30606	2.39029	-4.811804
**H**	-2.33181	2.710172	-4.649784
**C**	-0.509361	3.07276	-5.726508
**H**	-0.916721	3.919192	-6.269636
**C**	0.806704	2.668108	-5.94421
**H**	1.430007	3.198712	-6.655856
**C**	1.315363	1.576648	-5.246415
**H**	2.336953	1.253002	-5.413966
**C**	0.515062	0.889849	-4.33398
**H**	0.92418	0.038515	-3.801486
**C**	-0.664812	1.224409	-0.258622
**C**	-0.545191	2.44789	-0.97647
**H**	-0.445286	2.441823	-2.052932
**C**	-0.464384	3.655688	-0.334808
**H**	-0.335709	4.546981	-0.932987
**C**	-0.521078	3.746003	1.088895
**C**	-0.635635	2.521381	1.811838

**H**	-0.678501	2.52496	2.892022
**C**	-0.691482	1.318223	1.161521
**H**	-0.776469	0.420524	1.756465
**C**	-0.345861	6.185042	0.965794
**H**	0.593343	6.231079	0.40727
**H**	-0.371569	7.024235	1.65531
**H**	-1.178635	6.297165	0.267711
**C**	-0.469102	5.003271	3.186215
**H**	-1.398703	4.595745	3.593105
**H**	-0.391489	6.04163	3.496348
**H**	0.373807	4.45559	3.615744
**C**	-0.011237	-4.877188	-1.175768
**C**	-0.471789	-5.145274	-2.487316
**H**	-0.886788	-4.346526	-3.090609
**C**	-0.392645	-6.429335	-3.000141
**H**	-0.746838	-6.630863	-4.004759
**C**	0.141636	-7.463314	-2.22587
**H**	0.203562	-8.466558	-2.634088
**C**	0.597327	-7.212499	-0.926135
**H**	1.008872	-8.020466	-0.331544
**C**	0.523785	-5.93567	-0.401432
**H**	0.872192	-5.723073	0.601109
**C**	1.00313	-2.219621	3.108613
**H**	1.950738	-2.622652	2.750798

**H**	0.316561	-3.047138	3.282978
**C**	1.159748	-1.330491	4.302513
**C**	0.163023	-1.28118	5.28132
**H**	-0.719799	-1.907712	5.186349
**C**	0.301005	-0.447759	6.388484
**H**	-0.47279	-0.425018	7.148826
**C**	1.439301	0.345799	6.524008
**H**	1.553125	0.985059	7.393214
**C**	2.439885	0.301777	5.553004
**H**	3.334479	0.905875	5.665071
**C**	2.299786	-0.532811	4.448095
**H**	3.087139	-0.575898	3.700032

SI3 References

(1) Hursthouse, M. B.; Coles, S. J. The UK National Crystallography Service; Its Origins, Methods and Science. *Crystallogr. Rev.* **2014**, *20* (2), 117–154. https://doi.org/10.1080/0889311X.2014.884565.
(2) Sheldrick, G. M. SHELXT - Integrated Space-Group and Crystal-Structure Determination. *Acta Crystallogr. A* **2015**, *71* (1), 3–8. https://doi.org/10.1107/S2053273314026370.
(3) Farrugia, L. J. WinGX and ORTEP for Windows: An Update. *J. Appl. Crystallogr.* **2012**, *45* (4), 849–854. https://doi.org/10.1107/S0021889812029111.
(4) Zhao, Y.; Truhlar, D. G. The M06 Suite of Density Functionals for Main Group Thermochemistry, Thermochemical Kinetics, Noncovalent Interactions, Excited States, and Transition Elements: Two New Functionals and Systematic Testing of Four M06-Class Functionals and 12 Other Functionals. *Theor. Chem. Acc.* **2008**, *120* (1–3), 215–241. https://doi.org/10.1007/s00214-007-0310-x.
(5) Shao, Y.; Molnar, L. F.; Jung, Y.; Kussmann, J.; Ochsenfeld, C.; Brown, S. T.; Gilbert, A. T. B.; Slipchenko, L. V.; Levchenko, S. V.; O'Neill, D. P.; DiStasio, R. A., Jr.; Lochan, R. C.; Wang, T.; Beran, G. J. O.; Besley, N. A.; Herbert, J. M.; Lin, C. Y.; Van Voorhis, T.; Chien, S. H.; Sodt, A.; Steele, R. P.; Rassolov, V. A.; Maslen, P. E.; Korambath, P. P.; Adamson, R. D.; Austin, B.; Baker, J.; Byrd, E. F. C.; Dachsel, H.; Doerksen, R. J.; Dreuw, A.; Dunietz, B. D.; Dutoi, A. D.; Furlani, T. R.; Gwaltney, S. R.; Heyden, A.; Hirata, S.; Hsu, C.-P.; Kedziora, G.; Khalliulin, R. Z.; Klunzinger, P.; Lee, A. M.; Lee, M. S.; Liang, W.; Lotan, I.; Nair, N.; Peters, B.; Proynov, E. I.; Pieniazek, P. A.; Rhee, Y. M.; Ritchie, J.; Rosta, E.; Sherrill, C. D.; Simmonett, A. C.; Subotnik, J. E.; Woodcock, H. L., III; Zhang, W.; Bell, A. T.; Chakraborty, A. K.; Chipman, D. M.; Keil, F. J.; Warshel, A.; Hehre, W. J.; Schaefer, H. F., III; Kong, J.; Krylov, A. I.; Gill, P. M. W.; Head-Gordon, M. Advances in Methods and Algorithms in a Modern Quantum Chemistry Program Package. *Phys. Chem. Chem. Phys.* **2006**, *8* (27), 3172–3191. https://doi.org/10.1039/b517914a.
(6) Boys, S. F.; Bernardi, F. The Calculation of Small Molecular Interactions by the Differences of Separate Total Energies. Some Procedures with Reduced Errors (Reprinted from Molecular Physics, Vol 19, Pg 553-566, 1970). *Mol. Phys.* **2002**, *100* (1), 65–73. https://doi.org/10.1080/00268970110088901.
(7) Coropceanu, V.; Cornil, J.; da Silva Filho, D. A.; Olivier, Y.; Silbey, R.; Bredas, J.-L. Charge Transport in Organic Semiconductors. *Chem. Rev.* **2007**, *107* (4), 926–952. https://doi.org/10.1021/cr050140x.
(8) Bredas, J. L.; Beljonne, D.; Coropceanu, V.; Cornil, J. Charge-Transfer and Energy-Transfer Processes in Pi-Conjugated Oligomers and Polymers: A Molecular Picture. *Chem. Rev.* **2004**, *104* (11), 4971–5003. https://doi.org/10.1021/cr040084k.
(9) Nelsen, S. F.; Blackstock, S. C.; Kim, Y. Estimation of Inner Shell Marcus Terms for Amino Nitrogen Compounds by Molecular Orbital Calculations. *J. Am. Chem. Soc.* **1987**, *109* (3), 677–682. https://doi.org/10.1021/ja00237a007.
(10) Coropceanu, V.; Malagoli, M.; da Silva, D. A.; Gruhn, N. E.; Bill, T. G.; Bredas, J. L. Hole- and Electron-Vibrational Couplings in Oligoacene Crystals: Intramolecular Contributions. *Phys. Rev. Lett.* **2002**, *89* (27), 2755031–2755034. https://doi.org/10.1103/PhysRevLett.89.275503.
(11) Jensen, F. *Introduction to Computational Chemistry*; sons, J. W. and, Ed.; John Wiley and sons, 2007.

(12) Szabo, A.; Ostlund, N. S. *Modern Quantum Chemistry: Introduction to Advanced Electronic Structure Theory*; McGraw-Hill, 1989.
